# Transcranial stimulation of the developing brain: a plea for extreme caution

**DOI:** 10.3389/fnhum.2014.00600

**Published:** 2014-08-05

**Authors:** Nick J. Davis

**Affiliations:** Department of Psychology, Swansea UniversitySwansea, UK

**Keywords:** TMS, tDCS, non-invasive, neuroethics, safety, paediatric

## Introduction

Transcranial stimulation can be used to modulate the activity of the brain. Recent developments in our understanding of technologies such as transcranial magnetic or electrical stimulation have afforded reasonable grounds for optimism that techniques such as TMS or tDCS might be effective treatments for neurally-mediated disorders. Researchers have demonstrated encouraging benefits of TMS and tDCS in treating conditions such as tinnitus (Burger et al., 2011), depression (Arul-Anandam and Loo, 2009), and stroke (Nowak et al., 2010). Collectively these techniques are often referred to as “non-invasive brain stimulation” (NIBS), although I would argue that this term is not appropriate since in all cases energy is being transferred across the skull (Davis and van Koningsbruggen, 2013), and the use of this term may be misleading to the general public who are not aware of the documented risks associated with these procedures.

More recently it has been suggested that brain stimulation be used to treat neurological disorders in pediatric cases. A recent review by Vicario and Nitsche (2013a) identified a number of opportunities and challenges for the use of brain stimulation in children. Here I offer a plea for calm and for caution. The ethical stakes in clinical and research procedures with children are high enough that a conservative approach is warranted. Many of the ethical issues, relevant both to adult and child participants, have been touched on by other authors (e.g., Cohen Kadosh et al., 2012; Krause and Cohen Kadosh, 2013); however this paper will focus on the gaps in our knowledge that affect our ability to assess risk in translating brain stimulation procedures to pediatric cases.

There are a number of known risks associated with brain stimulation. Mild side-effects may include scalp tenderness, headache or dizziness, which are typically associated with the mechanism of delivery or with being immobilized in a chair or frame, and which may be under-reported (Brunoni et al., 2011). More serious effects may include seizure, mood changes or induction of hyper- or hypo-mania. However, the risk of seizure is low, at around 0.1% of adult cases and around 0.2% of pediatric reports, although these figures may not reflect unreported off-label use of the techniques (Rossi et al., 2009). These more serious symptoms are largely associated with people who already possess a degree of susceptibility, such as people with a history of epilepsy (Davis et al., 2013). Adult brain stimulation is thought be reasonably safe when used within defined limits (see below), however here I wish to focus on a number of factors that complicate the translation of TMS and tDCS protocols to pediatric cases.

I will focus on the key unknowns in brain stimulation research:

1. The unknown effects of stimulation;
2. The unknown side-effects of stimulation;
3. The lack of clear dosing guidelines;
4. The lack of translational studies from adults to children.

I will set out these “known unknowns” in translating our knowledge about TMS and tDCS effects to clinical pediatric applications, and touch on the practical and ethical barriers to their widespread usage.

## Gaps in our knowledge

### The unknown effects of stimulation

It is thought that the effects of stimulation on the brain involve modulating the excitability of cortical areas near to the tCS electrode or to the TMS coil. However, there are considerable gaps in our knowledge of how this modulation is achieved and maintained. It is assumed that long term depression- or potentiation-like processes mediate a change in the resting potential of neurons (e.g., Fritsch et al., 2010), and it is likely that the induced electric currents induce plastic changes in neurotransmitter availability (Stagg et al., 2009; Stagg and Nitsche, 2011), but the biophysical mechanism for the induction of these processes from electric fields is obscure. It is not clear to what extent white matter is involved in mediating the effects of brain stimulation. Children are known to show less myelination in some brain regions than adults (Klingberg et al., 1999; Barnea-Goraly et al., 2005), and it is thought that non-uniformity in brain tissue has a large role in determining the spread of current (Shahid et al., 2013). It is even less clear to what extent glial cells are involved during brain stimulation, although it is known that many of the changes in brain structure that occur during childhood and adolescence are due to changes in glial density (Caviness et al., 1996). These architectonic differences between child and adult brains are likely to affect the spread of applied current through brain tissue, making it more difficult to predict the electric field at, or away from, target brain areas.

### The unknown side-effects of stimulation

As well as the short-term effects of transcranial stimulation, we do not yet understand the effects of long-term use. It seems likely that repeated sessions of TMS or tCS lead to longer-lasting neural effects; these long-duration effects are what makes brain stimulation an attractive possibility for clinical treatment. However, no brain region exists in isolation, and researchers are only now beginning to understand the knock-on effects of modulating one brain area on other areas in the brain. For example, there is evidence that enhancing one aspect of cognition may be detrimental to other cognitive faculties, making neuromodulation a zero-sum intervention (Brem et al., 2014). Conversely, reduction in activation of a brain area may induce a paradoxical overall facilitation in function (Earp et al., 2014), through disinhibition in a network or through changes in neural noise. These notions suggest that we should be checking more widely for possible adverse effects of brain stimulation, since the resulting effect of stimulation may not be seen in the hypothesized behavior, but in behaviors governed elsewhere in a brain network. There is also the worrying possibility that electrical stimulation of the skull may induce or inhibit bone growth, an issue of particular importance in children whose cranial bones are not yet fused (Friedenberg et al., 1971, 1974). This latter possibility has not been explored in human volunteers in brain stimulation experiments.

### The lack of clear dosing guidelines

It is currently not known how to determine the appropriate dose of stimulation to give to an individual person to achieve a given size of effect. At present our best knowledge in dose-setting comes from studies that model the electric and magnetic fields generated in stimulation, and attempt to relate these fields to physical effects on brain tissue. For example, the current applied between two tDCS electrodes placed on the scalp induces an electric field across the brain surface (Miranda et al., 2006). Modeling this electric field may in principle afford predictions of the behavioral effect of specified levels of current (e.g., Mendonca et al., 2011). However, there are known to be considerable differences in the modeled field between individuals, depending on such factors as fat deposits, cortical folding and skull thickness. Importantly, one recent modeling study suggests that the transmission of electric current to the brain is more efficient in children than in adults, implying that clinicians should be more conservative in dose-setting for children than for adults (Kessler et al., 2013). This latter study suggested that the same electric field magnitude at the brain surface might be achieved with half of the applied current in children compared to adults. However, it is interesting to note that TMS-induced motor potentials are generated at a higher TMS intensity in children than older people, possibly as a result of different levels of inhibitory processing in the cortex (Mall et al., 2004). While not a complete solution, developing individual MRI-derived models for dose prediction is likely to remain the most effective strategy for safe delivery of brain stimulation.

### The lack of translational studies from adults to children

It is a well-established principle that children should not be considered as “small adults” when testing medical interventions. A recent study suggested that most medical devices used in children are never tested in pediatric populations before approval (Hwang et al., 2014). I argued above that modeling studies can inform our ability to safely apply the correct level of dose in individual children. However, we are left with an ethical dilemma: how to judge the safety of a procedure in children without exposing children to the procedure's potential risks during testing? This is not an uncommon problem in vulnerable groups. For example, in order to be certain that a drug is safe for use in pregnancy, it must be tested on pregnant women (Chambers et al., 2008). In the case of drug testing in pregnancy, this requires that physicians monitor and report rare adverse effects. Brain stimulation is similarly associated with rare and subtle side-effects, although in this case the patient may not be aware of or able to report these adverse effects. I propose that a clear system be developed for recording adverse effects in people with limited capacity to report these effects.

## Wider ethical concerns

We have seen how incomplete knowledge of the effects of brain stimulation in adults and in children may entail risks when applied to children, and have seen that TMS and tCS are likely to be of use in treating neurally-mediated disorders. In younger patients, the most promising treatment targets are epileptic disorders, depression and chronic pain, where some benefits have been shown in adults (Eldaief et al., 2013). There is at present a small number of publications that support the use of brain stimulation in developmental cognitive conditions including autism (Oberman et al., 2013; Enticott et al., 2014), attention deficit-hyperactivity disorder (e.g., Bloch et al., 2010) or developmental dyslexia (e.g., Costanzo et al., 2013; Vicario and Nitsche, 2013b).

Recently researchers have suggested that brain stimulation might enhance performance, in domains such as mathematical ability (Snowball et al., 2013), sport (Davis, 2013), moral reasoning (Young et al., 2010) and vigilance (Nelson et al., 2014). The possibility exists that a child might take a dose of stimulation before sitting an exam or a driving test. As access to brain stimulation becomes more widespread, in particular an internet-based do-it-yourself movement (“DIY-tDCS”), it is increasingly likely that people will take the findings reported in scientific reports and in the press, and attempt to apply the same stimulation parameters without the safeguards of the lab or clinic (Fitz and Reiner, 2013). Researchers and clinicians therefore have an increased duty of caution in presenting our findings to a wider audience.

## Conclusion

I have so far presented a somewhat negative view of the use of brain stimulation in younger people. In balance, I would add that based on the published literature, amounting to around 1000 pediatric cases, the protocols do not appear to expose patients to significantly enhanced risk of serious adverse effects. Adverse reactions have occurred, although generally these have been in patients who have an increased risk, such as in a case of rTMS leading to a seizure in a patient with elevated blood alcohol levels (Chiramberro et al., 2013). Sessions of TMS and tDCS are reasonably well tolerated in studies that have reported subjective experience. Rajpakse and Kirton (2013) and Krause and Cohen Kadosh (2013) give comprehensive recent overviews of brain stimulation studies in children. When used with care, brain stimulation in children appears to be safe and well tolerated, at least over the range of expected effects that occur following stimulation.

I therefore hope to offer a positive conclusion. Transcranial stimulation will almost certainly play a large role in future treatment options for neurological disorders in children, including the developmental cognitive disorders listed above, for which there is some theoretical justification for optimism. Many of the disorders discussed here are disorders of plasticity; the hope is that maladapted communication between or within brain areas might be adjusted through the use of externally-applied stimulation. Certainly in adults TMS and tDCS are likely to be associated with fewer and less unpleasant side-effects than the neuroactive drugs that they are intended to replace, and brain stimulation is thought to be safe when used within known safety parameters (e.g., Green et al., 1997; Bikson et al., 2009; Rossi et al., 2009; Davis et al., 2013).

It is clear that a large amount still remains to be done in establishing safe use of brain stimulation for children. The major practical problems that remain are: safe dosing of stimulation for individual children; developing a framework for establishing informed consent in children and their guardians; and an efficient system for monitoring and reporting adverse effects during and following brain stimulation in minors. Researchers and clinicians should also be conscious that children and parents are increasingly technologically aware, and that headline-grabbing news related to brain stimulation could lead people to self-administer stimulation; this is already occurring, as a brief search of internet forums will reveal.

Brain stimulation is a powerful tool, and it is our duty to ensure that it is used responsibly in people who are most vulnerable. With scientific and practical developments, we can be confident that brain stimulation offers an opportunity to help those who have most to benefit.

### Conflict of interest statement

The author declares that the research was conducted in the absence of any commercial or financial relationships that could be construed as a potential conflict of interest.
